# Multi-breed genomic prediction using Bayes R with sequence data and dropping variants with a small effect

**DOI:** 10.1186/s12711-017-0347-9

**Published:** 2017-09-21

**Authors:** Irene van den Berg, Phil J. Bowman, Iona M. MacLeod, Ben J. Hayes, Tingting Wang, Sunduimijid Bolormaa, Mike E. Goddard

**Affiliations:** 1 Faculty of Veterinary and Agricultural Science, University of Melbourne, Parkville, VIC Australia; 2 Agriculture Victoria, AgriBio, Centre for AgriBioscience, Bundoora, VIC 3083 Australia; 3 School of Applied Systems Biology, La Trobe University, Bundoora, VIC 3083 Australia; 4 Queensland Alliance for Agriculture and Food Innovation, Centre for Animal Science, University of Queensland, St Lucia, QLD Australia

## Abstract

**Background:**

The increasing availability of whole-genome sequence data is expected to increase the accuracy of genomic prediction. However, results from simulation studies and analysis of real data do not always show an increase in accuracy from sequence data compared to high-density (HD) single nucleotide polymorphism (SNP) chip genotypes. In addition, the sheer number of variants makes analysis of all variants and accurate estimation of all effects computationally challenging. Our objective was to find a strategy to approximate the analysis of whole-sequence data with a Bayesian variable selection model. Using a simulated dataset, we applied a Bayes R hybrid model to analyse whole-sequence data, test the effect of dropping a proportion of variants during the analysis, and test how the analysis can be split into separate analyses per chromosome to reduce the elapsed computing time. We also investigated the effect of imputation errors on prediction accuracy. Subsequently, we applied the approach to a dataset that contained imputed sequences and records for production and fertility traits for 38,492 Holstein, Jersey, Australian Red and crossbred bulls and cows.

**Results:**

With the simulated dataset, we found that prediction accuracy was highly increased for a breed that was not represented in the training population for sequence data compared to HD SNP data. Either dropping part of the variants during the analysis or splitting the analysis into separate analyses per chromosome decreased accuracy compared to analysing whole-sequence data. First, dropping variants from each chromosome and reanalysing the retained variants together resulted in an accuracy similar to that obtained when analysing whole-sequence data. Adding imputation errors decreased prediction accuracy, especially for errors in the validation population. With real data, using sequence variants resulted in accuracies that were similar to those obtained with the HD SNPs.

**Conclusions:**

We present an efficient approach to approximate analysis of whole-sequence data with a Bayesian variable selection model. The lack of increase in prediction accuracy when applied to real data could be due to imputation errors, which demonstrates the importance of developing more accurate methods of imputation or directly genotyping sequence variants that have a major effect in the prediction equation.

**Electronic supplementary material:**

The online version of this article (doi:10.1186/s12711-017-0347-9) contains supplementary material, which is available to authorized users.

## Background

The increasing availability of whole-sequence data, which should contain causative mutations for complex traits, is expected to increase the accuracy of genomic prediction and to aid in the identification of these causative mutations. There are two advantages of using sequence data over single nucleotide polymorphism (SNP) chip genotypes. First, if the SNP chip does not explain all of the genetic variance explained by the sequence, prediction accuracy will be limited regardless of the prediction method used. Second, if there is no single SNP that is in complete linkage disequilibrium (LD) with a quantitative trait locus (QTL), prediction accuracy using SNP chip genotypes will decrease. In particular, the latter influences Bayesian prediction methods, which work best when they identify a single SNP with a large effect. Both of these reasons concern the LD between causative mutations and SNPs. In dairy cattle, LD is extensive within a breed but the phase of LD varies between breeds [1], which is expected to decrease across-breed prediction. Use of sequence data is expected to increase the accuracy of multi-breed and across-breed prediction, which would be beneficial for breeds with small reference population sizes [2].

However, results from both simulation studies and analysis of real data do not always show an increase in accuracy from sequence data compared to SNP chip genotypes. The large number of variants makes analysis of all sequence variants and accurate estimation of all effects computationally challenging. Furthermore, the higher rate of genotype errors due to imputation errors in sequence data compared to SNP chip data [3], may limit the benefit of sequence data over SNP chips. Studies using whole-sequence data in dairy cattle [4] and chicken [5] showed no or very little increase in prediction accuracy compared to high-density SNP data, using either genomic best linear unbiased prediction (GBLUP) or a Bayesian variable selection model. Several stimulation studies [6, 7] indicate that, rather than analysing all sequence variants together, preselecting variants that are close to the causative mutations can lead to increased prediction accuracy. In dairy cattle [8, 9] and *Drosophila* [10], substantial increases in accuracy were obtained when several tens, hundreds or thousands variants were selected based on a genome-wide association study (GWAS) and used for prediction in addition to genome-wide SNPs.

On the contrary, other studies show that preselecting sequence variants can lead to an increase in bias and, thus, an increase in accuracy is not evident. Calus et al. [11] used split-and-merge Bayesian selection, where the analysis was split into several subsets that were analysed in a first step to select the most informative variants. Subsequently, selected variants were analysed together. This resulted in a prediction accuracy that is slightly lower or equal to that obtained with the 50 K SNP chip, and increased the bias. Similar results were obtained by Veerkamp [12], using a conditional and joint GWAS. Both Calus et al. [11] and Veerkamp et al. [12] used data on one breed only, Holstein, and the long distance over which LD is conserved within Holstein populations [1] reduces the potential benefit of sequence data over medium- or high-density SNP data [13]. Another approach is preselection of variants based on their functional annotations, which results in small increases in accuracy in dairy cattle [14] and chickens [15], although Heidaritabar et al. [5] found no increases in prediction accuracy using a similar approach in chickens.

While promising results were obtained by selecting variants based on a GWAS [9], it required testing a large number of scenarios to find a set of variants that increased prediction accuracy. Furthermore, because a GWAS generally tests only one SNP at a time, it does not account for LD between SNPs, which results in the selection of many variants associated with the same QTL. Limiting the number of variants per QTL resulted in a higher accuracy than selecting all variants with a *p* value below a certain threshold. Therefore, a model that analyses multiple SNPs simultaneously may be more efficient in identifying sequence variants that increase prediction accuracy than a GWAS, which tests SNPs one at a time. Bayesian variable selection models are effective for the identification of causative mutations [16]. However, analysing all sequence variants simultaneously is computationally expensive. To speed up the analysis, Wang et al. [17] developed a hybrid version of the Bayes R variable selection model, which substantially decreases the computing time by first running an expectation–maximization (EM) module, followed by a reduced number of Monte Carlo Markov chain (MCMC) iterations. To further decrease computing time, a proportion of the variants can be dropped either directly after the EM module, or after a number of MCMC iterations.

While the Bayes R hybrid model decreases computing time substantially compared to Bayes R [17], estimating effects for millions of sequence variants simultaneously remains computationally challenging. An approximation to analysing all sequence variants simultaneously could be achieved by splitting up the analysis per chromosome, which makes it feasible to analyse all variants on a chromosome with a Bayesian variable selection model, such as the Bayes R hybrid model.

Our objective was to find a strategy to approximate multi-breed and across-breed prediction, by analysing whole-sequence data with a Bayesian variable selection model. First, we used a simulated dataset that consisted of a filtered set of whole-genome sequence variants to test the accuracy of the Bayes R hybrid model. We also considered the effect of dropping variants with little or no effect during the analysis and tested how the analysis can be split into chromosomes to reduce the elapsed computing time. Furthermore, we investigated the effect of imputation errors on the prediction accuracy. Subsequently, we applied the tested approach to a dataset that contains imputed sequences and records for production and fertility traits for a large number of Holstein, Jersey, Australian Red and crossbred bulls and cows.

## Methods

For this study, we used two datasets: a small dataset, with a reduced number of variants and simulated phenotypes, to speed up initial comparisons of different scenarios and a second dataset to test the scenarios in practice, which contained a much larger number of sequence variants and individuals, with daughter trait deviations (DTD) for bulls and trait deviations (TD) for cows for milk, fat, protein and fertility.

### Simulated data

The simulated dataset was the AUS-Sim set that is described in more detail by Macleod et al. [14]. This dataset consisted of realised imputed sequence variants for 3047 Holstein bulls, 4942 Holstein cows, 770 Jersey bulls, 1553 Jersey cows, 869 Red Holstein bulls, 741 Australian Red cows and 114 Australian Red bulls. All Holstein and Jersey individuals were used as reference population and the Red Holstein and Australian Red individuals as validation population.

All individuals were genotyped with the Illumina BovineSNP50 chip [18], or custom 50 K chips, and either genotyped with or imputed to the 800 K Illumina BovineHD beadChip. For part of the analysis, the 600,641 SNPs on the HD chip were used (HD). In addition, genotypes for approximately two million sequence variants in gene coding regions and variants that were 5000 bp up- and down-stream of genes were imputed. Annotations for the sequence variants were collated using NGS-SNP [19]. After filtering out variants with a minor allele frequency (MAF) lower than 0.0002 and variants in complete LD, this dataset (SEQ) contained 994,019 variants, including 45,026 non-synonymous coding (NSC) variants, 578,734 variants located within 5 kb upstream and downstream of genes, or in 3/5′ untranslated genic regions (REG), and 370,259 variants on the HD chip.

QTL were randomly sampled from all SEQ variants. In total, 4000 causative mutations were simulated, of which 3485, 500 and 15 were categorised as having small, medium and large effects on the trait. Effects were sampled from three normal distributions, with a mean of 0 and variances of 0.0001$\sigma_{g}^{2}$, 0.001$\sigma_{g}^{2}$ and 0.01$\sigma_{g}^{2}$ for small, medium and large QTL respectively, where $\sigma_{g}^{2}$ is the additive genetic variance. The true breeding value (TBV) of individual $j$ was computed as $TBV_{j} = \sum\nolimits_{i = 1}^{4000} {x_{ij} a_{i} }$, where $x_{ij}$ is the genotype of individual $j$ for QTL $i$, and $a_{i}$ the additive effect of QTL $i$. To obtain a phenotype with a heritability ($h^{2}$) of 0.6, an environmental effect was sampled from a normal distribution and added to the TBV. A Holstein breed effect was sampled from $N$(10, 1) and added to the phenotype for all Holstein individuals.

To investigate the effect of imputation errors on prediction accuracy, errors were added to the SEQ variants for the reference population, the validation population or both populations. For each allele, the probability of an error ($e$) was simulated as $e = \frac{r}{{\sqrt {MAF} }}$, where $r$ was equal to 0.0013, 0.0027, 0.0066, 0.0132 or 0.0264 to simulate an average $e$ of 0.005, 0.0101, 0.025, 0.050 or 0.100, respectively. Each imputation error scenario was replicated 10 times.

Pedigree information for all individuals was obtained from the Australian Dairy Herd Improvement Scheme (ADHIS) and Interbull.

### Real data

The second dataset contained daughter trait deviations (DTD) or trait deviations (TD) for milk, fat, protein and fertility for 38,540 animals. Animals were genotyped with the Illumina BovineSNP50 chip [18] and imputed to or directly genotyped with the Illumina 800 K BovineHD bead chip. Subsequently, sequences of Holstein, Jersey and Australian Red bulls and cows from Run 5 of the 1000 bulls genome project [20] were used as the reference set to impute sequence genotypes for all individuals using FImpute [21]. During the imputation process, FImpute failed to impute parts of chromosomes 12 and 23, and for these regions, only the HD genotypes were available. This was the case between 25 and 30 Mb on chromosome 12 and between 62 and 70.5 Mb and between 72.5 and 75 Mb on chromosome 23. These regions contained a large number of structural variants and had a low density of HD SNPs, which may have hindered the imputation process. After imputation, the dataset contained 21,379,438 variants, of which 90,010 NSC, 1459,566 REG, 5520,343 intronic variants, 77,299 synonymous variants and 14,232,221 intergenic variants. The HD SNP chip contained 3977 and 360,816 of the synonymous and intergenic variants, respectively. The number of variants used for the analysis was substantially smaller after removing variants with a MAF lower than 0.002 and LD pruning. LD pruning was performed using PLINK [22] to remove variants in high LD (r^2^ > 0.9). For LD pruning, variants were divided into four groups based on their functional annotations: NSC variants, REG variants, variants on the HD chip and all other variants. Annotations for the sequence variants were collated using the NGS-SNP software [19]. LD pruning was first performed within each group, followed by removal of REG variants with an r^2^ higher than 0.9 with a NSC variant, HD variants with an r^2^ higher than 0.9 with a NSC variant or a REG variant and other variants with an r^2^ higher than 0.9 with a NSC variant. After filtering based on MAF and LD, 4812,745 variants were retained for further analysis.

The dataset was split up into a reference population with Holstein and Jersey bulls born before 2005, and Holstein, Jersey and crossbred cows, and a validation population with Holstein and Jersey bulls born in 2005 and after, and Australian Red bulls and cows. Animals in the reference population that had sons in the validation population were removed from the dataset. Furthermore, seven animals were removed from the dataset because their sequence differed for less than 10,000 variants from another individual in the dataset. Because of the presence of crossbred individuals, a principal component analysis (PCA) was used to divide the Holstein and Jersey animals in five different clusters, as shown in Figure S1 (see Additional file 1: Figure S1). Clusters 1, 2 and 3 contained mainly Holstein individuals, while clusters 4 and 5 contained mainly Jersey individuals. The crossbred individuals were present in all clusters. Three Jersey cows were removed from the analysis because they were assigned to clusters 1 and 2, and one Holstein cow and one Holstein bull were removed from the analysis because they were assigned to cluster 5. The clusters were set as fixed effect to account for breed differences. In total, the reference population for production traits contained 35,775 individuals, including 22,868 Holstein cows, 3124 Holstein bulls, 6144 Jersey cows, 787 Jersey bulls and 2852 crossbred cows. An overview of the reference population is in Table 1. In the validation population, the number of individuals in clusters 3 and 4 was small, i.e. 28 and 20 individuals, respectively. Therefore, the individuals in these clusters were not used in the analysis. In total, the validation population contained 2717 individuals, including 799 Holstein bulls, 200 Jersey bulls, 1579 Australian Red cows and 139 Australian Red bulls. Table 2 summarizes the validation population.

**Table 1:** Reference population

Cluster	Breed	Sex	Production	Fertility
1	Holstein	Cows	8757	7853
	Holstein	Bulls	1246	1230
	Crossbred	Cows	447	401
2	Holstein	Cows	12,140	10,926
	Holstein	Bulls	1607	1551
	Crossbred	Cows	824	735
3	Holstein	Cows	1936	1831
	Holstein	Bulls	271	229
	Jersey	Cows	10	10
	Jersey	Bulls	1	1
	Crossbred	Cows	738	684
4	Holstein	Cows	35	30
	Jersey	Cows	609	584
	Jersey	Bulls	190	145
	Crossbred	Cows	710	668
5	Jersey	Cows	5525	5281
	Jersey	Bulls	596	551
	Crossbred	Cows	133	109

The number of individuals in the reference population is split up per cluster, breed and sex for production traits and fertility

**Table 2:** Number of individuals in the validation population

Cluster	Breed	Sex	Production	Fertility
1	Holstein	Bulls	357	294
2	Holstein	Bulls	442	338
5	Jersey	Bulls	200	167
–	Australian Red	Cows	1579	1507
–	Australian Red	Bulls	139	133

The number of individuals in the validation population is split up per cluster, breed and sex for production traits and fertility

### Statistical analysis

We used the hybrid version of the Bayes R mixture model described by Wang et al. [23] for our analyses:

$${\mathbf{y}} = {\mathbf{Xb}} + {\mathbf{Za}} + {\mathbf{Wv}} + {\mathbf{e}} ,$$

where ${\mathbf{y}}$ is a vector of phenotypes (TD or DTD), ${\mathbf{X}}$ a design matrix that allocates phenotypes to vector ${\mathbf{b}}$ with fixed effects, fitting the overall mean, breed and sex as fixed effects, ${\mathbf{Z}}$ is a design matrix that allocates phenotypes to vector ${\mathbf{a}}$ with polygenic breeding values distributed as $N(0,{\mathbf{A}}\sigma_{a}^{2}$), where ${\mathbf{A}}$ the pedigree-based relationship matrix, $\sigma_{a}^{2}$ is the polygenic variance, ${\mathbf{W}}$ is a design matrix of genotypes, ${\mathbf{v}}$ a vector of variant effects, and ${\mathbf{e}}$ a vector of residual errors distributed as $N(0,{\mathbf{E}}\sigma_{e}^{2}$), where ${\mathbf{E}}$ is a diagonal matrix with diagonals $1/w_{j}$, where the weighting coefficient $w_{j}$ is based on the number of records available for individual $j$ [24], and $\sigma_{e}^{2}$ is the residual variance. Variant effects (${\mathbf{v}}$) were drawn from one of four normal distributions with $N(0,0\sigma_{g}^{2}$), $N(0,0001\sigma_{g}^{2}$), $N(0,001\sigma_{g}^{2}$), and $N(0,01\sigma_{g}^{2}$), respectively, where $\sigma_{g}^{2}$ is the additive genetic variance. The prior distribution for the proportion of variants in each of these distributions was ${\mathbf{P}}\sim{\text{Dirichlet}}\left( {\varvec{\upalpha}} \right)$, ${\varvec{\upalpha}} = \left[ {1,1,1,1} \right]$.

The hybrid variant of Bayes R uses first an expectation–maximization (EM) module to estimate ${\mathbf{a}}$, ${\mathbf{P}}$, ${\mathbf{b}}$, ${\mathbf{v}}$, and $\sigma_{e}^{2}$. Then, the estimates of these parameters are used as starting values for the subsequent Monte Carlo Marcov chain (MCMC) module, for 10,000 iterations, without burn-in.

The accuracy of prediction was defined as the correlation of the predicted breeding value with the TD (cows) or DTD (bulls) between validation animals.

### Dropping of variants

To speed up the analysis, it is possible to drop some of the variants during the different stages of analysis (e.g. after the EM step or after a certain number of MCMC iterations). Variants were ranked based on their posterior inclusion probability (PIP) to be included in any of the distributions with a non-zero variance, and the variants with the lowest PIP were dropped in order to drop the desired proportion of variants. After dropping, the mixing proportions at the time of dropping were added to the prior for the rest of the analysis, to account for the dropped variants.

### Scenarios in the simulated dataset

Using the simulated dataset, we tested several strategies to analyse sequence data, which are summarized in Table 3: all sequence variants analysed together (S_FULL_D0), all variants analysed per chromosome (S_CHR_D*d*), variants selected based on their PIP from each chromosome (CHR) reanalysed with all chromosomes together (S_KEPT_D*d*), and variants selected by CHR and all HD variants reanalysed with all chromosomes together (S + HD_KEPT + HD_D*d*). As a comparison to the S_FULL_D0 scenario, we analysed all HD genotypes (HD_FULL_D0). In the S_FULL_D*d* scenarios, the sequence variants were analysed simultaneously with *d* = 0, 0.25, 0.5, 0.7 or 0.9 as the target proportion of variants dropped during the analysis. Variants were dropped after the EM step, after 200 MCMC iterations, or after 10,000 MCMC iterations.

**Table 3:** Overview of scenarios

Scenario	Data	Strategy	DropIter	DropProp	Simulation	Real
HD_FULL_D0	HD	FULL	–	0	Y	Y
S_FULL_D0	SEQ	FULL	–	0	Y	N
S_FULL_D0.25	SEQ	FULL	0, 200 or 10,000	0.25	Y	N
S_FULL_D0.50	SEQ	FULL	0, 200 or 10,000	0.50	Y	N
S_FULL_D0.7	SEQ	FULL	0, 200 or 10,000	0.70	Y	N
S_FULL_D0.9	SEQ	FULL	0, 200 or 10,000	0.90	Y	N
S_CHR_D0	SEQ	CHR	0	0	Y	N
S_CHR_D0.7	SEQ	CHR	10,000	0.70	Y	N
S_CHR_D0.9	SEQ	CHR	10,000	0.90	Y	N
S_KEPT_D0.7	SEQ	KEPT	10,000	0.70	Y	N
S_KEPT_D0.9	SEQ	KEPT	10,000	0.90	Y	Y
S + HD_KEPT + HD_D0.7	SEQ + HD	KEPT + HD	10,000	0.70	Y	N
S + HD_KEPT + HD_D0.9	SEQ + HD	KEPT + HD	10,000	0.90	Y	N

HD = HD genotypes used for prediction, S = sequence variants used for prediction, FULL = all variants analysed together, CHR = all variants analysed per chromosome, KEPT = variants selected by CHR reanalysed with all chromosomes together, KEPT + HD = variants selected by CHR and all HD variants reanalysed with all chromosomes together, dropProp = proportion of variants that is dropped after dropIter MCMC iterations, simulation and real indicate whether the scenario was analysed in the simulated and real datasets

In scenarios S_CHR_D*d* with *d* = 0, 0.7 or 0.9, the sequence variants were split up and analysed per chromosome. The effects of variants that were estimated during HD_FULL_D0 were used to correct the DTD and TD for all other chromosomes except the chromosome that was analysed. After analysing all the chromosomes, the estimated effects of variants of all the chromosomes were used to estimate a genome-wide breeding value.

Using the variant effects estimated by S_CHR_D*d* directly to compute breeding values assumes that effects are estimated independently between chromosomes. Therefore, in scenarios S_KEPT_D*d* with *d* = 0.7 or 0.9, variants that were retained in the model by S_CHR_D*d* were reanalysed in a genome-wide analysis to re-estimate effects of variants and GEBV.

The approach used in scenarios S_KEPT + HD_D*d*, with *d* = 0.7 or 0.9, was the same as S_KEPT_D*d¸* except that in addition to the variants that were retained in the model for the analyses per chromosome, the HD variants were added into the model.

For scenarios HD_FULL_D0, S_FULL_D*d* and S_CHR_D*d*, the prior for the number of variants per distribution was ${\varvec{\upalpha}} = \left[ {1,1,1,1} \right]$, whereas for S_KEPT_D*d* and S_KEPT + HD_D*d*, this was set to the posterior estimate of the number of variants per distribution obtained by S_FULL_D*d*.

### Scenarios with the real data

Using the real dataset, we compared scenarios HD_FULL_D0, S_CHR_D0.9, S_KEPT_D0.9 and S_KEPT + HD_D0.9. For HD_FULL_D0, the prior for the number of variant per distribution was ${\varvec{\upalpha}} = \left[ {1,1,1,1} \right]$, and the posterior estimate of the HD_FULL_D0 scenario was used as prior for S_CHR_D0.9 and S_KEPT_D0.9.

### Animal ethics statement

No ethical approval was required for this study.

## Results

### Simulation

**Fig. 1:**
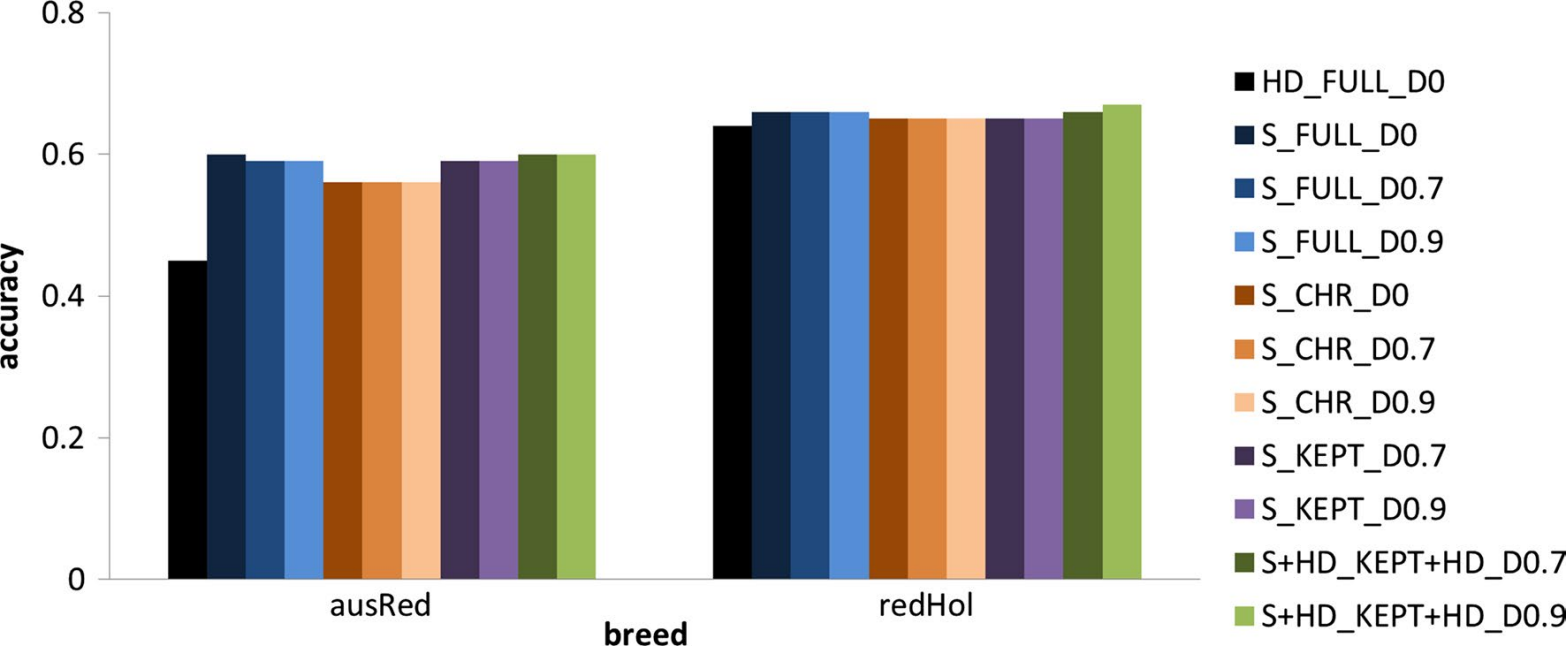
Accuracy of different strategies using the simulated dataset. Analysing all HD variants together without dropping any variants (HD_FULL_D0), analysing all sequence variants together while dropping 0% (S_FULL_D0), 70% (S_FULL_D0.7) or 90% (S_FULL_D0.9) of the variants after 10,000 MCMC iterations, analysing sequence variants per chromosome while dropping 0% (S_CHR_D0), 70% (S_CHR_D0.7) or 90% (S_CHR_D0.9) of the variants after 10,000 MCMC iterations, variants selected by S_CHR_D0.7 and S_CHR_D0.9 reanalysed with all chromosomes together (S_KEPT_D0.7 and S_KEPT_D0.9), or variants selected by S_CHR_D0.7 and S_CHR_D0.9 and all HD variants reanalysed with all chromosomes together (S + HD_KEPT + HD_D0.7 and S + HD_KEPT + HD_D0.9); ausRed = Australian Red, redHol = Red Holstein

**Fig. 2:**
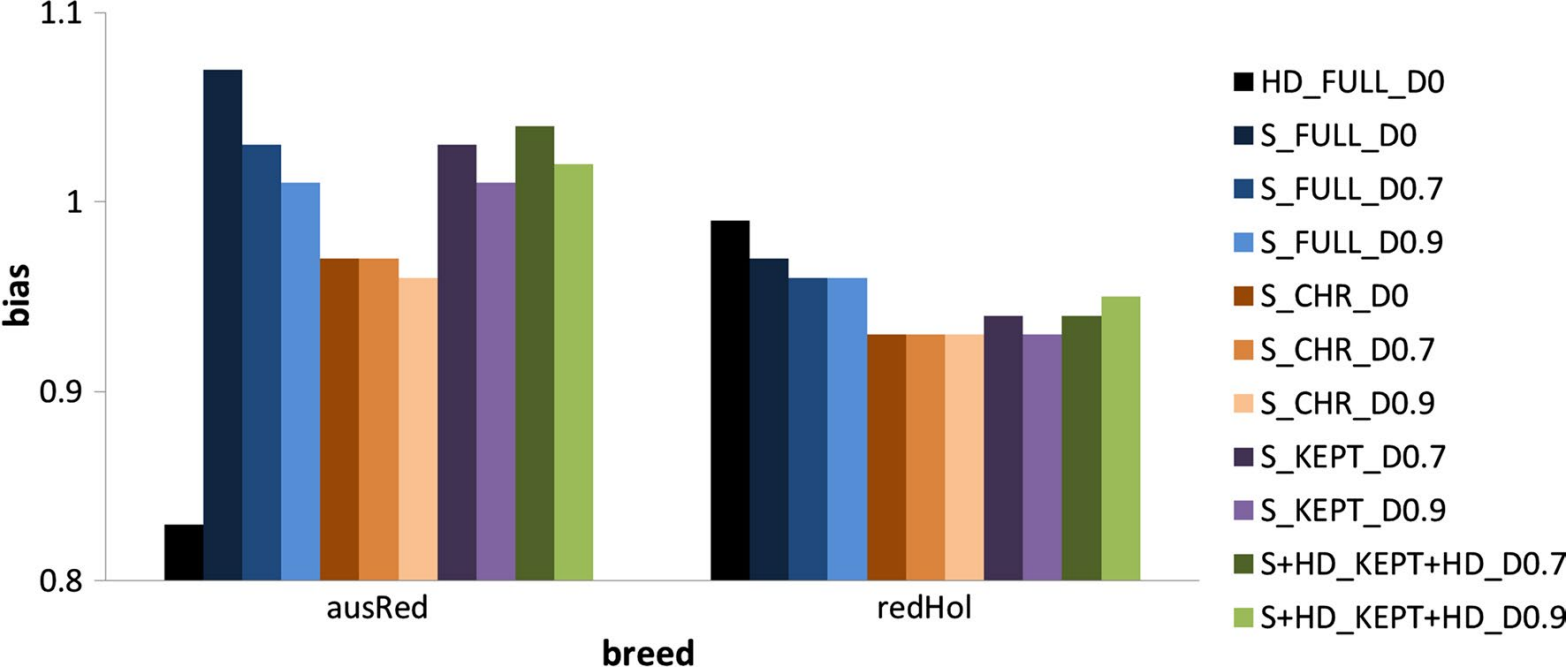
Bias of different strategies using the simulated dataset. Analysing all HD variants together without dropping any variants (HD_FULL_D0), analysing all sequence variants together while dropping 0% (S_FULL_D0), 70% (S_FULL_D0.7) or 90% (S_FULL_D0.9) of the variants after 10,000 MCMC iterations, analysing sequence variants per chromosome while dropping 0% (S_CHR_D0), 70% (S_CHR_D0.7) or 90% (S_CHR_D0.9) of the variants after 10,000 MCMC iterations, variants selected by S_CHR_D0.7 and S_CHR_D0.9 reanalysed with all chromosomes together (S_KEPT_D0.7 and S_KEPT_D0.9), or variants selected by S_CHR_D0.7 and S_CHR_D0.9 and all HD variants reanalysed with all chromosomes together (S + HD_KEPT + HD_D0.7 and S + HD_KEPT + HD_D0.9); ausRed = Australian Red, redHol = Red Holstein

**Fig. 3:**
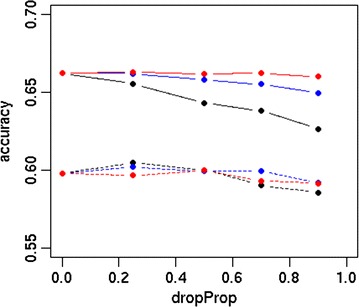
Prediction accuracy as a function of the proportion of dropped variants. Variants were dropped after EM (black), 200 MCMC iterations (blue) or 10,000 MCMC iterations (red), line = Red Holstein, dashed line = Australian Red, dropProp = proportion of dropped variants

**Fig. 4:**
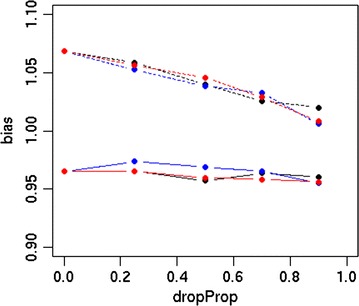
Bias as a function of the proportion of dropped variants. Variants were dropped after EM (black), 200 MCMC iterations (blue) or 10,000 MCMC iterations (red), continuous line = Red Holstein, dashed line = Australian Red, dropProp = proportion of dropped variants

The accuracy and bias of the different strategies are in Figs. 1 and 2, respectively. Differences between scenarios were more pronounced for Australian Red than for Red Holstein. For both breeds, the accuracy was higher using sequence data than HD data. The S_FULL_D0 scenario resulted in accuracies of 0.60 and 0.66 for Australian Red and Red Holstein individuals, respectively, while HD_FULL_D0 yielded accuracies of 0.45 and 0.64. Dropping 70 or 90% of the variants after 10,000 MCMC iterations resulted in accuracies that were similar or slightly reduced compared to those with S_FULL_D0. Dropping variants directly after the EM module or after 200 MCMC iterations decreased accuracy, as shown in Fig. 3. Accuracy decreased as the proportion of dropped variants increased and increased as the number of MCMC iterations increased before deciding which variants to drop. Figure 4 shows the bias as a function of the proportion of dropped variants. There was no consistent increase or decrease in bias across breeds when more variants were dropped.

Splitting up the analyses per chromosome and analysing all chromosomes in parallel decreased the computing time from 55 h for S_FULL_D0 to between 1.9 and 4.5 h per chromosome. However, the accuracy was lower than that obtained by S_FULL_D0. The reduction in accuracy was up to 0.04 in Australian Red and 0.01 in Red Holstein. Combining the process of dropping 70 or 90% of the variants with splitting up the analysis per chromosome did not decrease accuracy furthermore.

Contrary to the S_CHR_D*d* scenarios, reanalysing the variants that were kept in the model in a genome-wide analysis in the S_KEPT_D*d* scenarios resulted in accuracies that were similar or only slightly lower than those obtained with S_FULL_D*d*. Adding the HD variants in the S_KEPT + HD_D*d* scenarios resulted in accuracies that were similar to those obtained with S_FULL_D*d*.

Table 4 compares the number of variants assigned to each of the four distributions for the different scenarios. Generally, sequence data resulted in a larger number of variants with effects drawn from the distributions with small and large variances compared to HD data. Compared to the number of simulated QTL (3485 small, 500 medium and 15 large QTL), the number of variants included in these distributions tended to be overestimated, especially the number of variants with effects drawn from the distribution with the largest variance. While only 15 QTL had an effect size that corresponded to the distribution with the largest variance, the number of variants assigned to this distribution varied from 21 for HD_FULL_D0 to 71 for S_CHR_D0. Overestimation of the number of variants in the fourth distribution was largest in the S_CHR_D0 scenario.

**Table 4:** Average number of variants per distribution over the number of iterations in the simulated dataset

Data	Analysis	Drop	Number of variants per distribution			
			0 $\sigma_{g}^{2}$	0.0001 $\sigma_{g}^{2}$	0.001 $\sigma_{g}^{2}$	0.01 $\sigma_{g}^{2}$
HD	FULL	0.0	592,931	3286	898	21
S	FULL	0.0	914,767	5053	666	48
		0.7	369,322	2464	471	43
		0.9	172,007	1350	407	42
S	CHR	0.0	915,665	4279	519	71
		0.7	371,643	2279	396	65
		0.9	171,554	1350	358	61
S	KEPT	0.7	298,238	2118	499	44
		0.9	98,494	908	390	45
S + HD	KEPT + HD	0.7	759,835	4459	663	44
		0.9	650,252	3813	616	45

HD = HD genotypes used for prediction, S = sequence variants used for prediction, FULL = all variants analysed together, CHR = all variants analysed per chromosome, KEPT = variants selected by CHR reanalysed with all chromosomes together, KEPT + HD = variants selected by CHR and all HD variants reanalysed with all chromosomes together, dropProp = proportion of variants that is dropped after 10,000 MCMC iterations, $\sigma_{g}^{2}$ = additive genetic variance

Table 5 shows the proportion of variance explained by prediction markers ($h_{M}^{2}$) and the polygenic component ($h_{A}^{2}$), and the heritability computed as: $h^{2} = h_{M}^{2} + h_{A}^{2}$. In the HD_FULL_D0 scenario, $h^{2}$ was equal to 0.59 and thus, was close to the simulated heritability of 0.60. In the scenarios using sequence data, $h^{2}$ was highest when all sequence variants were used (0.64). When variants were dropped, $h_{A}^{2}$ increased slightly, while $h^{2}$ and $h_{M}^{2}$ decreased. In the S_KEPT_D0.7 scenario, $h^{2}$, $h_{M}^{2}$ and $h_{A}^{2}$ were equal to 0.60, 0.59 and 0.01, respectively. The largest $h_{A}^{2}$ i.e. 0.05 was obtained with the S_KEPT_D0.9 scenario. The highest $h^{2}$ were obtained with the S_KEPT + HD_D*d* scenarios, i.e. 0.65 and 0.63 for S_KEPT + HD_D0.7 and S_KEPT + HD_D0.9, respectively.

**Table 5:** Proportion of variance explained by markers ($h_{M}^{2}$) and polygenic effect ($h_{A}^{2}$) in the simulated dataset

Data	Analysis	Drop	$h_{M}^{2}$	$h_{A}^{2}$	$h^{2}$
HD	FULL	0.0	0.57	0.02	0.59
S	FULL	0.0	0.63	0.01	0.64
		0.7	0.60	0.02	0.62
		0.9	0.58	0.02	0.60
S	KEPT	0.7	0.59	0.01	0.60
		0.9	0.52	0.05	0.57
S + HD	KEPT + HD	0.7	0.64	0.01	0.65
		0.9	0.62	0.01	0.63

$h^{2} = h_{M}^{2} + h_{A}^{2}$, HD = HD genotypes used for prediction, S = sequence variants used for prediction, FULL = all variants analysed together, CHR = all variants analysed per chromosome, KEPT = variants selected by CHR reanalysed with all chromosomes together, KEPT + HD = variants selected by CHR and all HD variants reanalysed with all chromosomes together, dropProp = proportion of variants that is dropped after 10,000 MCMC iterations

Table 6 shows the number of simulated QTL dropped or retained with their respective posterior inclusion probability (PIP). For all scenarios, the majority of QTL had a PIP between 0 and 0.01. In the scenarios in which variants were dropped, the majority of QTL were dropped, and the number of dropped QTL increased as the proportion of dropped variants increased. The number of QTL in the classes with a PIP higher than 0.01 varied between scenarios. The number of variants with a PIP between 0.5 and 1 was largest in the S_CHR_D*d* scenarios.

**Table 6:** Number of simulated QTL dropped or retained with their respective posterior inclusion probability (PIP)

Analysis	Drop	Dropped	PIP					
			0–0.01	0.01–0.05	0.05–0.1	0.1–0.2	0.2–0.5	0.5–1
FULL	0.0	0	3368	435	40	15	19	20
	0.7	2159	1179	463	35	19	19	23
	0.9	2981	293	520	36	24	18	25
CHR	0.0	0	3337	465	36	12	21	26
	0.7	2177	1164	460	36	14	20	26
	0.9	3025	306	461	42	15	19	29
KEPT	0.7	2177	1088	531	38	21	22	20
	0.9	3025	251	514	41	22	19	25
KEPT+	0.7	2177	1193	446	32	17	15	17
HD	0.9	3025	347	440	34	18	16	17

FULL = all variants analysed together, CHR = all variants analysed per chromosome, KEPT = variants selected by CHR reanalysed with all chromosomes together, KEPT + HD = variants selected by CHR and all HD variants reanalysed with all chromosomes together, drop = proportion of variants that are dropped after 10,000 MCMC iterations

**Fig. 5:**
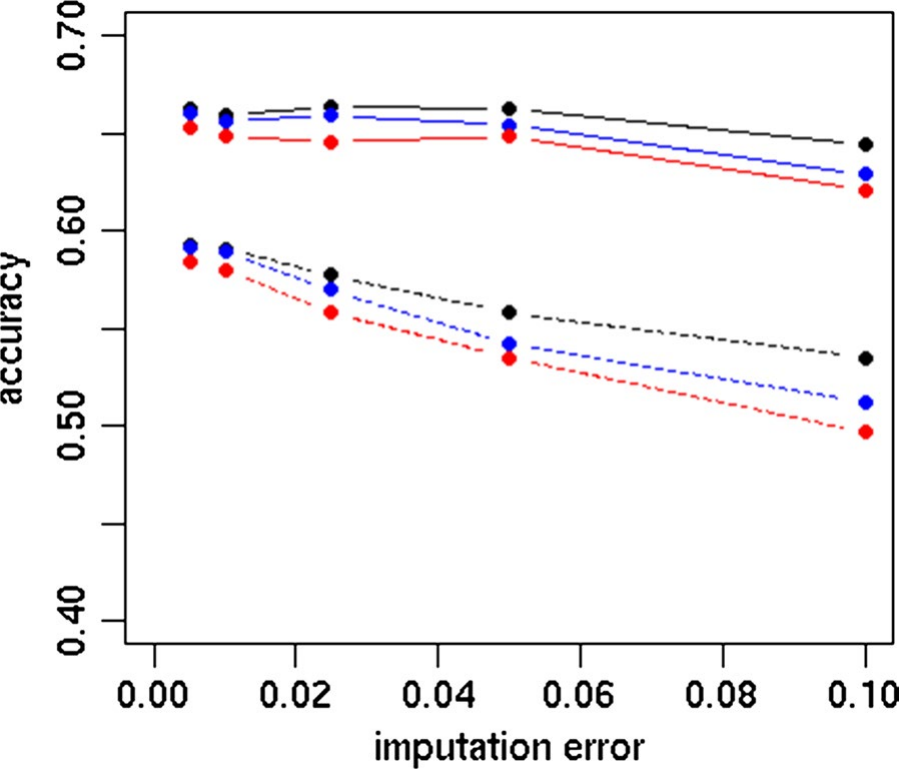
Prediction accuracy as a function of imputation error. Imputation errors were added to both reference and validation population (black), only the reference population (blue) or only the validation population (red), continuous line = Red Holstein, dashed line = Australian Red

**Fig. 6:**
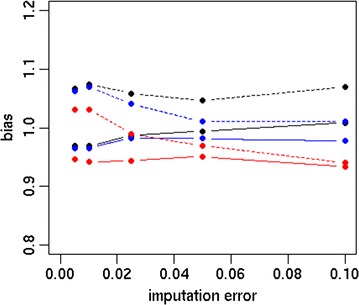
Bias as a function of imputation error. Imputation errors were added to both reference and validation populations (black), only the reference population (blue) or only the validation population (red), continuous line = Red Holstein, dashed line = Australian Red

Figures 5 and 6 show the prediction accuracy and bias as a function of the imputation error. The prediction accuracy decreased as the number of imputation errors increased but there was no clear pattern for bias and this decrease was larger for Australian Red than for Red Holstein. It was larger when imputation errors were added only to the validation population than when they were added to the training population or to both the training and validation populations.

### Real data

**Fig. 7:**
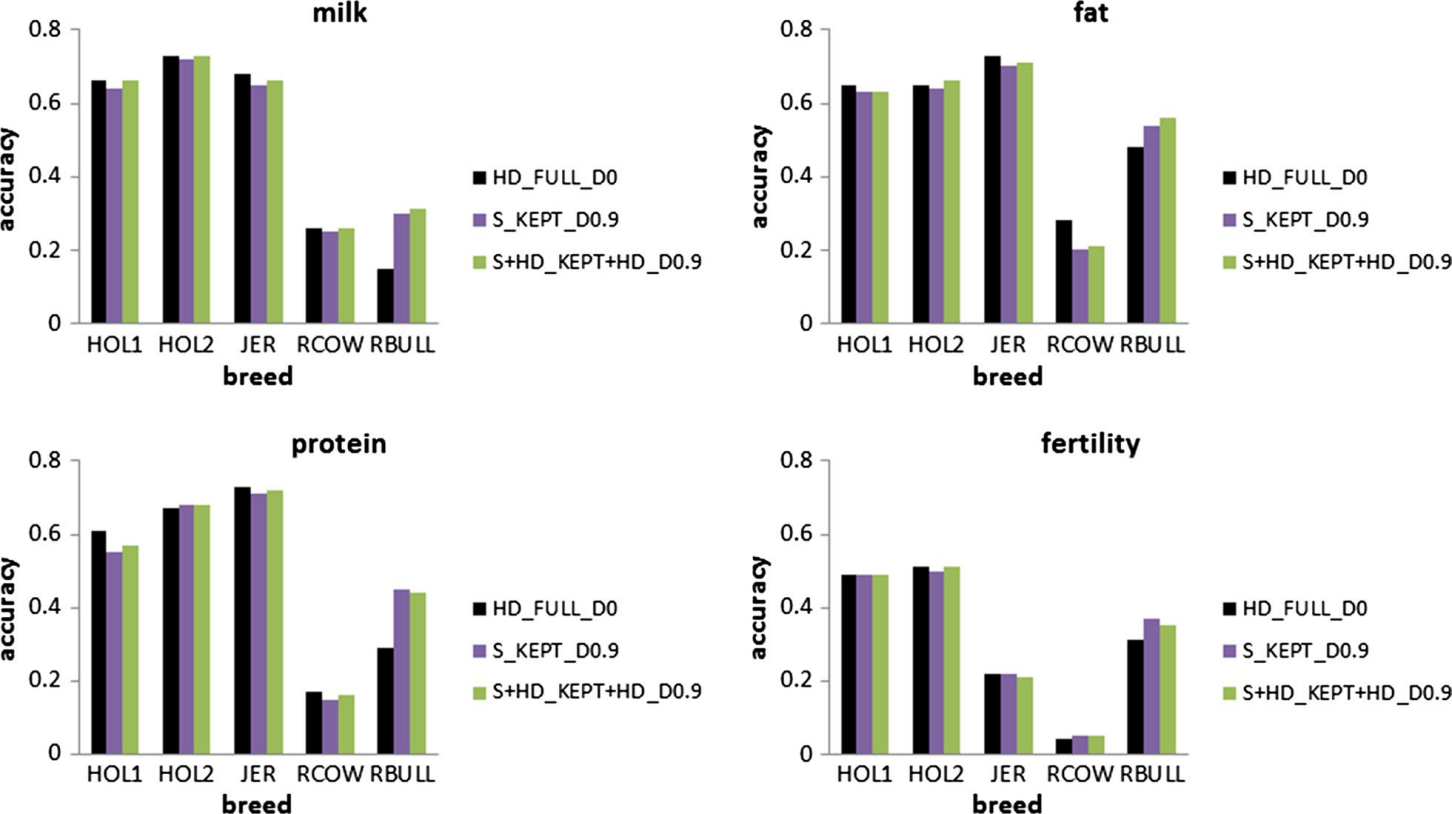
Accuracy of different scenarios using real data. Analysing all HD variants together without dropping any variants (HD_FULL_D0), dropping 90% of the variants per chromosome and reanalysing the remaining variants with all chromosomes together (S_KEPT_D0.9), or dropping 90% of variants per chromosome and reanalysing the remaining variants and all HD variants with all chromosomes together (S + HD_KEPT + HD_D0.9); HOL1 = Holstein cluster 1, HOL2 = Holstein cluster 2, JER = Jersey, RCOW = Australian Red cows, RBULL = Australian Red bulls

**Fig. 8:**
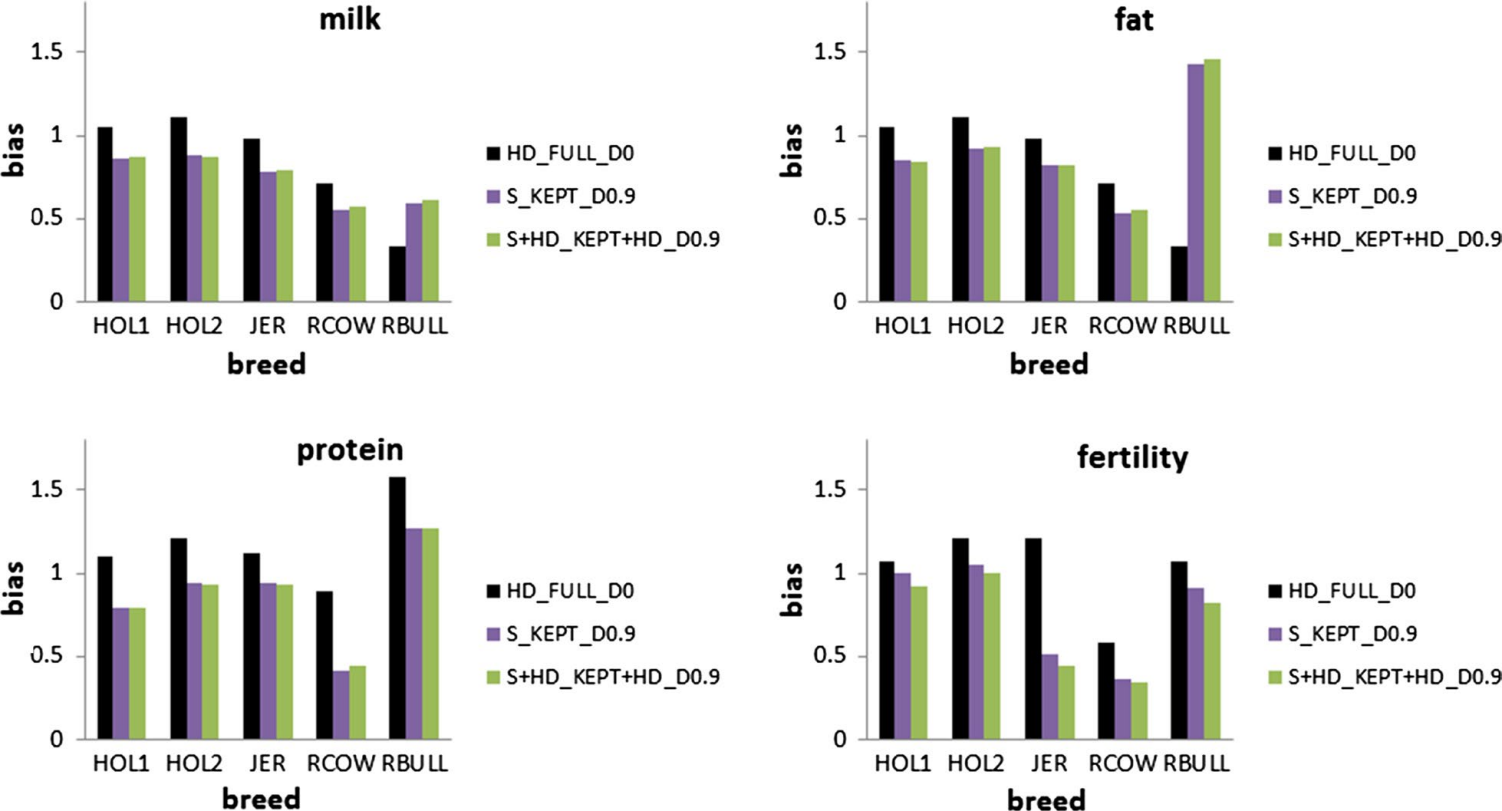
Bias of different scenarios using real data. Analysing all HD variants together without dropping any variants (HD_FULL_D0), dropping 90% of the variants per chromosome and reanalysing the remaining variants with all chromosomes together (S_KEPT_D0.9), or dropping 90% of variants per chromosome and reanalysing the remaining variants and all HD variants with all chromosomes together (S + HD_KEPT + HD_D0.9), HOL1 = Holstein cluster 1, HOL2 = Holstein cluster 2, JER = Jersey, RCOW = Australian Red Cows, RBULL = Australian Red Bulls

The accuracy and bias of the scenarios tested with real data are in Figs. 7 and 8. For all traits, S_KEPT_D0.9 and S_KEPT + HD_D0.9 tended to result in reduced accuracy and increased bias compared to HD_FULL_D0. Sequence data resulted in substantially increased accuracies only for Australian Red Bulls. Holstein bulls were grouped in two clusters, and accuracies were higher for the bulls in the HOL2 cluster that was closest to Jersey individuals in the PCA. Averaged across traits, the difference in accuracy of the S_KEPT_D0.9 scenario compared to the HD_FULL_D0 scenario was equal to −0.03, −0.01, −0.02, −0.03 and 0.11 for HOL1, HOL2, JER, RCOW and RBULL, respectively. Adding the HD variants improved the accuracy slightly with, averaged across traits, a difference compared to HD_FULL_D0 of −0.02, 0.01, −0.02, −0.02 and 0.11 for HOL1, HOL2, JER, RCOW and RBULL, respectively. Decreases in accuracy were smallest for fertility and largest for fat yield. The bias of the prediction was larger with the S_KEPT_D0.9 and S_KEPT + HD_D0.9 scenarios than with HD_FULL_D0 for HOL1, JER and RCOW. For HOL2, the bias was similar in all three scenarios, although with HD_FULL_D0, regression coefficients were above 1, while for S_KEPT_D0.9 and S_KEPT + HD_D0.9, regression coefficients were below 1. For RBULL, the bias was large for all scenarios and not consistently better in any one.

The number of variants assigned to each of the four distributions is in Table 7. S_KEPT_D0.9 resulted in fewer variants in the distribution with zero effect, more variants in the distribution with a small variance, and generally fewer or a similar number of variants in the distributions with medium and large variances. In the S_KEPT + HD_D0.9 scenario, there were more variants in both the distributions with zero effect and with a small variance than in the other scenarios, and generally fewer or a similar number of variants in the distribution with medium and large variances. Contrary to the milk production traits, for fertility, both S_KEPT_D0.9 and S_KEPT + HD_D0.9 resulted in more variants in the distribution with a medium variance than HD_FULL_D0.

**Table 7:** Average number of variants per distribution over the number of iterations in the real dataset

Trait	Data	Analysis	DropProp	Number of variants per distribution			
				0 $\sigma_{g}^{2}$	0.0001 $\sigma_{g}^{2}$	0.001 $\sigma_{g}^{2}$	0.01 $\sigma_{g}^{2}$
Milk	HD	FULL	0	627,503	4299	22	10
	S	KEPT	0.9	483,521	6603	17	6
	S + HD	KEPT + HD	0.9	1076,643	8927	22	6
Fat	HD	FULL	0	627,510	4312	9	4
	S	KEPT	0.9	483,614	6307	7	4
	S + HD	KEPT + HD	0.9	1080,078	8890	9	3
Prot	HD	FULL	0	627,347	4476	9	3
	S	KEPT	0.9	482,957	6352	8	3
	S + HD	KEPT + HD	0.9	1078,647	9025	9	2
Fert	HD	FULL	0	625,899	5668	260	8
	S	KEPT	0.9	548,382	5715	310	5
	S + HD	KEPT + HD	0.9	1135,712	10,569	436	7

Prot = protein, fert = fertility, HD = HD genotypes used for prediction, S = sequence variants used for prediction, FULL = all variants analysed together, KEPT = variants selected per chromosome reanalysed with all chromosomes together, KEPT + HD = variants selected per chromosome and all HD variants reanalysed with all chromosomes together, dropProp = proportion of variants dropped after 10,000 MCMC iterations, $\sigma_{g}^{2}$ = additive genetic variance

Table 8 shows $h_{M}^{2}$, $h_{A}^{2}$ and $h^{2}$ obtained with real data. $h_{M}^{2}$ and $h^{2}$ were lowest in the HD_FULL_D0 scenario and highest in the S_KEPT + HD_D0.9 scenario, and $h_{A}^{2}$ was highest in the HD_FULL_D0 scenario and lowest in the S_KEPT + HD_D0.9 scenario. For milk production traits, differences between S_KEPT + HD_D0.9 and HD_FULL_D0 varied between 0.22 and 0.24 for $h_{M}^{2}$, 0.10 and 0.12 for $h^{2}$, and were equal to 0.12 for $h_{A}^{2}$. Differences between S_KEPT + HD_D0.9 and S_KEPT_D0.9 were smaller, varying between 0.04 and 0.05 for $h_{M}^{2}$, 0.03 and 0.05 for $h^{2}$, and were equal to −0.01 for $h_{A}^{2}$. For fertility, $h^{2}$ was much lower, which resulted in smaller differences between scenarios, although the overall trend was the same as for production traits.

**Table 8:** Proportion of variance explained by prediction markers ($h_{M}^{2}$) and polygenic effect ($h_{A}^{2}$)

Trait	Data	Analysis	DropProp	$h_{M}^{2}$	$h_{A}^{2}$	$h^{2}$
Milk	HD	FULL	0.0	0.29	0.16	0.45
	S	KEPT	0.9	0.49	0.05	0.53
	S + HD	KEPT + HD	0.9	0.53	0.04	0.57
Fat	HD	FULL	0.0	0.22	0.15	0.37
	S	KEPT	0.9	0.39	0.04	0.42
	S + HD	KEPT + HD	0.9	0.44	0.03	0.47
Protein	HD	FULL	0.0	0.21	0.16	0.37
	S	KEPT	0.9	0.39	0.05	0.43
	S + HD	KEPT + HD	0.9	0.44	0.04	0.48
Fertility	HD	FULL	0.0	0.02	0.00	0.02
	S	KEPT	0.9	0.03	0.00	0.03
	S + HD	KEPT + HD	0.9	0.04	0.00	0.04

$h^{2} = h_{M}^{2} + h_{A}^{2}$, HD = high density SNP, HD = HD genotypes used for prediction, S = sequence variants used for prediction, FULL = all variants analysed together, KEPT = variants selected per chromosome reanalysed with all chromosomes together, KEPT + HD = variants selected per chromosome and all HD variants reanalysed with all chromosomes together, drop = proportion of variants dropped after 10,000 MCMC iterations

## Discussion

We focus the discussion on two points, i.e. (1) on the ability to reduce the computing time needed for analysis of whole-genome sequence data by using an EM-MCMC hybrid approach, dropping some variants from the analysis and processing chromosomes in parallel, and (2) on the reasons why genome sequence data may or may not result in higher accuracies than HD SNP genotypes.

### Approximate analysis of full sequence data with Bayes R

The simulated datasets were previously analysed by Macleod et al. [14], using Bayes R. We obtained the same accuracy using full sequence data with the hybrid version of Bayes R. This is in agreement with Wang et al. [23] who show that the accuracy with the hybrid model was equal to that with Bayes R, which confirms that the hybrid model is an efficient alternative to Bayes R.

We tested a new option of the hybrid model, which drops a proportion of the variants during the analysis to decrease the required computing time even more. The dropping of variants was tested at different stages of the analysis, and the proportion of variants that were dropped varied. While dropping variants reduced computing time, it resulted in a decrease in accuracy. The decrease in accuracy became smaller as fewer variants were dropped, and when variants were dropped after a large number of MCMC iterations. However, the goal of dropping variants is to reduce computing time, and the gain in computing time is smaller when fewer variants are dropped. Running the full MCMC chain before dropping any variants resulted in an accuracy that was similar to that in the analyses that did not drop any variants. However, if the analysis was run first for 10,000 iterations before dropping the variants, and subsequently run for another 10,000 iterations with the dropped variants computing time increased rather than decreased compared to analysing all the variants for 10,000 iterations without dropping any variants. Therefore, if the goal is to increase the speed of the analysis, it is better to use all the variants. However, if it is necessary to select variants that are associated with the trait, the results of the hybrid model can be used to select variants, but a large number of MCMC iterations is advisable. We note that dropping variants from the analysis can lead to bias. We prevented this by recording the mixing proportions for the four distributions immediately before any SNPs were dropped and adding this to the prior.

Analysing a few millions of sequence variants simultaneously is computationally challenging and would take a long time to complete. Therefore, we tested if it is possible to split up the analysis per chromosome. However, using the effects of SNPs estimated per chromosome directly to estimate breeding values resulted in a decreased accuracy compared to S_FULL_D*d*. Our approach is somewhat similar to that tested by Calus et al. [11]. Calus et al. [11] split up the variants, but in their approach, the LD between variants in a subset was minimized. By splitting up the analysis per chromosome, we maximised the LD. Calus et al. [11] observed that the performance of the model decreased when subsets contained variants in very high LD. This could explain why we found a reduced accuracy for the S_CHR_D*d* scenarios compared to S_FULL_D*d*, although we tried to address this issue by pruning out variants in high LD with each other. Furthermore, the dataset used by Calus et al. [11] contained only Holstein individuals, while our dataset contained individuals from multiple breeds, and LD is conserved over much longer distances within breeds than across breeds [1]. Our approach differs from that described by Calus et al. [11], in that we used the HD estimated effects to correct for all chromosomes except the chromosome being analysed. This would be the same as analysing full sequence data if the prediction based on full sequence data for other chromosomes was the same as the prediction based on HD SNPs. It appears that since the analysis based on sequence data changes the estimated effects of sequence variants, it is necessary to analyse the retained variants from all chromosomes together to maximise accuracy. Therefore, the analyses per chromosome were used to select variants rather than directly to predict breeding values. Rerunning the selected variants from all the chromosomes combined together increased the accuracy to a value that was equal or close to that obtained with S_FULL_D*d* in the simulation. However, this required to drop a large number of variants, which resulted in a decrease in accuracy even for the S_FULL_D*d* scenarios. The vast majority of variants that were dropped would probably have very small effects, and therefore were not likely to be linked to major QTL. They could, however, be used to explain part of the polygenic effects. Therefore, we added the HD variants to the analysis, which further increased the accuracy.

### Potential advantage of sequence data over HD SNP genotypes

Using the simulated data, analysis of sequence data resulted in a higher accuracy than analysis of HD SNP genotypes, i.e. there was a large advantage of S_FULL_D0 over HD_FULL_D0, and consequently, the accuracy of any scenario using sequence data was higher than HD_FULL_D0, even for the scenarios with an accuracy lower than that of S_FULL_D0. For the Red Holstein validation population, the advantage of S_FULL_D0 over HD_FULL_D0 was much smaller than for the Australian Red validation population. This is likely because the Red Holstein is much more closely related to the Holstein individuals in the reference populations. Because LD is conserved over much shorter distances across breeds than within breeds, sequence data is thought to be especially beneficial for multi-breed and across-breed prediction [2].

There are two reasons for the use of sequence data resulting in higher accuracy: it might capture more of the genetic variance and it might include QTL with large effects when there are no HD SNPs in complete LD with these QTL. However, the variance not explained by SNPs ($h_{A}^{2}$) was only 0.01 to 0.02 higher than when sequence data was analysed. Therefore, this does not explain the large increase in accuracy observed, and it appears that the prediction equation based on HD SNPs used SNPs in LD with the QTL and that the phase of LD differed in the validation and training populations. By comparison, the prediction based on sequence data must have emphasised variants that were closer to the QTL (or even were the QTL) and this LD was better conserved in the validation population. In reality, the missing heritability in HD SNP chip data is likely to be higher than 0.02, and consequently, the analysis of simulated data may underestimate the advantage of sequence data in this respect. Indeed, in the analysis of the real data, $h_{A}^{2}$ was much higher than in the simulation study. In the S_KEPT_D0.9 and S_KEPT + HD_D0.9 scenarios using real data, $h_{M}^{2}$ and $h^{2}$ were substantially higher than in HD_FULL_D0, while $h_{A}^{2}$ was lower, which suggests that using sequence data reduced the amount of missing heritability.

### Difference between results obtained with simulated and real data

When real data was analysed, sequence data resulted in an accuracy that was similar to that of HD_FULL_D0. This is in line with several studies that reported little or no advantage of sequence data over HD or 50 K genotypes, especially within breed [4, 11, 12], but it differs from results obtained in our simulation study. These differences may have been caused by differences between the simulated and real data. In the simulated data, we simulated a moderate number of QTL, which were present in the sequence data but not in HD data. In the real data, it is possible that the number of QTL was larger but that fewer QTL had medium to large effects, which made it more difficult for Bayes R to distinguish between variants in high LD with the causative mutations and variants that have no effect on the trait. In the simulated data, we assumed that all causative mutations had the same effect in all breeds, but in reality, breed x QTL interactions may result in different effects. In addition, not all sequence variants were included in the data analysed and it is likely that some causal mutations were absent. Furthermore, while an Australian Red validation population was used in both the simulated and real data, the Red Holstein bulls used as validation population were more distantly related to the Holstein individuals in the reference population than the Holstein bulls used as validation in the real dataset. Sequence data is expected to be more advantageous for multi-breed and across-breed prediction than for within-breed prediction, and therefore, using two relatively distantly related validation populations likely resulted in the sequence data to be more advantageous in the simulated dataset than in the within-breed scenarios.

Another potential cause of lack of accuracy in prediction using sequence data is that most sequence data are obtained by imputation rather than direct sequencing, and consequently, imputation errors are introduced. Because the genotypes used in the simulated dataset were obtained by imputation, it is likely that the imputation errors in this dataset are similar to those in the real dataset. However, in the simulation, the estimation of the effects of the causative mutations was based on the imputed genotypes, while in reality, the effects are based on the true genotypes. Therefore, the effect of imputation errors in the real data is expected to be larger. To investigate this, additional imputation errors were simulated, either in all individuals, only in the training population or only in the validation population. As expected, increasing the number of imputation errors decreased the accuracy, and the largest decrease in accuracy was observed when the errors were present in the validation population. In the training population, the effect of imputation errors is likely less marked, because the genotype errors can differ between individuals, and if the genotype is correct in the majority of animals, it may not have a large influence on the estimated effect. In contrast, errors in the genotypes of the validation population directly influence their estimated breeding value, and thereby the accuracy. For a few chromosomes, the correlation and concordance rate between imputed and true sequence genotypes were computed (see Additional file 2: Table S1). The expected reduction in prediction accuracy based on these correlations and concordance rates, is even greater than the observed reduction in accuracy in the scenarios using sequence data compared to HD_FULL_D0.

The correlation ranged from 0.92 for chromosome 5 to 0.94 for chromosomes 1 and 20, and the concordance from 0.94 to 0.95 (see Additional file 2: Table S1). While some imputation software programs provide a measure of imputation accuracy for each variant, this is not the case for FImpute, and we only filtered variants based on MAF. Filtering out incorrectly imputed variants may increase prediction accuracy.

In the real data, the only large increase in accuracy with sequence data was observed for Australian Red bulls. Because LD is conserved over shorter distances across breeds than within breeds [1], sequence data is expected to be especially beneficial for across-breed prediction [7]. However, for the Australian Red cows, the accuracy of the scenarios using sequence data was at most similar to that using HD data. While the vast majority of the Australian Red bulls were genotyped at HD, most cows were genotyped at lower densities. Consequently, imputation accuracy may be lower for the cows than for the bulls, which could be a possible explanation for the reduced accuracy observed in the Australian Red cows.

## Conclusions

We present an efficient approach to approximate analysis of full sequence data with a Bayesian variable selection model. While the simulation study provided promising results, when we applied the method to a real dataset, the accuracy obtained was at most similar to that obtained with HD genotypes, and bias increased. The lack of increase in prediction accuracy could be due to errors introduced in the genotypes by imputation. Therefore, it is necessary to develop more accurate methods of imputation or to directly genotype sequence variants that have an important effect in the prediction equation.

## Additional files

**Additional file 1: Figure S1.** Principal component analysis of Holstein and Jersey individuals. Description: *PC1* principal component 1, *PC2* principal component 2. Left graph shows the different clusters based on PC1 (dark blue = HOL1, medium blue = HOL2, light blue = HOL3, dark green = JER1, light green = JER2), right graph breeds based on the pedigree (dark blue = purebred Holstein, medium blue crossbreds with more Holstein than Jersey ancestors, light blue = Holstein × Jersey crossbreds, dark green = crossbreds with more Jersey than Holstein ancestors, light green = purebred Jersey).

**Additional file 2: Table S1.** Correlation and concordance between true and imputed sequence genotypes for variants on chromosomes 1, 5, 20 and 25.
