# Supplementary figures and images for: Multi-breed genomic prediction using Bayes R with sequence data and dropping variants with a small effect
Source: Genet Sel Evol. 2017 Sep 21;49:70. doi: 10.1186/s12711-017-0347-9 (PMC5609075; doi:10.1186/s12711-017-0347-9)

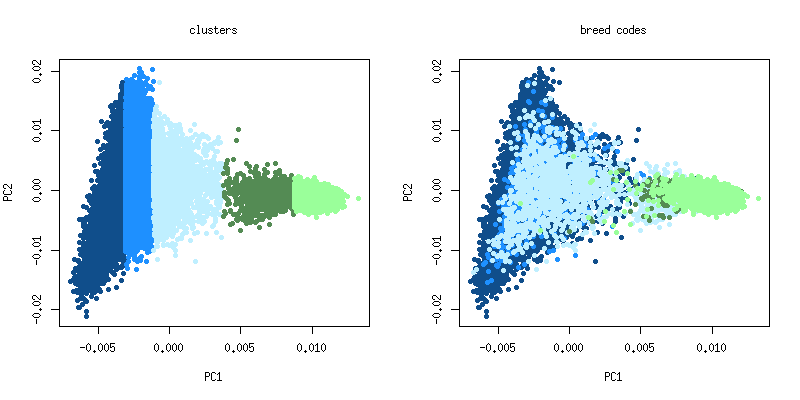

Supplement: Supplementary file 1 — Additional file 1: Figure S1. Principal component analysis of Holstein and Jersey individuals. Description: PC1 principal component 1, PC2 principal component 2. Left graph shows the different clusters based on PC1 (dark blue = HOL1, medium blue = HOL2, light blue = HOL3, dark green = JER1, light green = JER2), right graph breeds based on the pedigree (dark blue = purebred Holstein, medium blue crossbreds with more Holstein than Jersey ancestors, light blue = Holstein × Jersey crossbreds, dark green = crossbreds with more Jersey than Holstein ancestors, light green = purebred Jersey). [file 12711_2017_347_MOESM1_ESM.png]
