# Supplementary material for: Multi-breed genomic prediction using Bayes R with sequence data and dropping variants with a small effect
Source: Genet Sel Evol. 2017 Sep 21;49:70. doi: 10.1186/s12711-017-0347-9 (PMC5609075; doi:10.1186/s12711-017-0347-9)
Supplement: Supplementary file 2 — Additional file 2: Table S1. Correlation and concordance between true and imputed sequence genotypes for variants on chromosomes 1, 5, 20 and 25. [file 12711_2017_347_MOESM2_ESM.docx]

**Additional file 2: Table S1. Correlation and concordance between true and imputed sequence genotypes for variants on chromosome 1, 5, 20 and 25.**

| **Chromosome** | **Correlation** | **Concordance** |
| --- | --- | --- |
| 1 | 0.94 | 0.96 |
| 5 | 0.92 | 0.94 |
| 20 | 0.94 | 0.96 |
| 25 | 0.93 | 0.95 |
